# Spin structure factors of doped monolayer Germanene in the presence of spin-orbit coupling

**DOI:** 10.1038/s41598-021-87268-x

**Published:** 2021-04-07

**Authors:** Farshad Azizi, Hamed Rezania

**Affiliations:** Department of Physics, Razi University, Kermanshah, Iran

**Keywords:** Nanoscale materials, Electronic properties and materials

## Abstract

In this paper, we present a Kane-Mele model in the presence of magnetic field and next nearest neighbors hopping amplitudes for investigations of the spin susceptibilities of Germanene layer. Green’s function approach has been implemented to find the behavior of dynamical spin susceptibilities of Germanene layer within linear response theoryand in the presence of magnetic field and spin-orbit coupling at finite temperature. Our results show the magnetic excitation mode for both longitudinal and transverse components of spin tends to higher frequencies with spin-orbit coupling strength. Moreover the frequency positions of sharp peaks in longitudinal dynamical spin susceptibility are not affected by variation of magnetic field while the peaks in transverse dynamical susceptibility moves to lower frequencies with magnetic field. The effects of electron doping on frequency behaviors of spin susceptibilities have been addressed in details. Finally the temperature dependence of static spin structure factors due to the effects of spin-orbit coupling, magnetic field and chemical potential has been studied.

## Introduction

A lot of theoretical and experimental studies have been performed on Graphene as a one-atom-thick layer of graphite since it’s fabrication^1^. The low energy linear dispersion and chiral property of carbon structure leads to map the nearest neighbor hopping tight binding hamiltonian which at low energy to a relativistic Dirac Hamiltonian for massless fermions with Fermi velocity $v_{F}$. Novel electronic properties have been exhibited by Graphene layer with a zero band gap which compared to materials with a non-zero energy gap. These materials have intriguing physical properties and numerous potential practical applications in optoelectronics and sensors^2^.

Recently, the hybrid systems consisting of Graphene and various two-dimensional materials have been studied extensively both experimentally and theoretically^3–5^. Also, 2D materials could be used for a extensive applications in nanotechnology^6,7^ and memory technology^8^. While the research interest in Graphene-based superlattices is growing rapidly, people have started to question whether the Graphene could be replaced by its close relatives, such as 2 dimensional hexagonal crystal of Germanene. This material shows a zero gap semiconductor with massless fermion charge carriers since their $\pi$ and $\pi ^{*}$ bands are also linear at the Fermi level^9^. Germanene as counterpart of Graphene, is predicted to have a geometry with low-buckled honeycomb structure for its most stable structures in contrast to the Graphene monolayer^9,10^. Such small buckling as vertical distance between two planes of atoms for Germanene comes from the mixing of $sp^{2}$ and $sp^{3}$ hybridization^11,12^. The behavior of Germanene electronic structure shows a linear dispersion close to K and K’ points of the first Brillouin zone. However *ab initio* calculations indicated that spin-orbit coupling in Germanene causes to small band gap opening at the Dirac point and thus the Germanene has massive Dirac fermions^10,13^. Also the band gap due to the spin orbit coupling in Germanene is more remarkable rather than that in Graphene^14^. The intrinsic carrier mobility of Germanene is higher than Graphene^15^. The different dopants within the Germanene layer gives arise to the sizable band gap opening at the Dirac point an the electronic properties of this material are affected by that^16,17^. In a theoretical work, the structural and electronic properties of superlattices made with alternate stacking of Germanene layer are systematically investigated by using a density functional theory with the van der Waals correction^18^. It was predicted that spin orbit coupling and exchange field together open a nontrivial bulk gap in Graphene like structures leading to the quantum spin hall effect^19,20^. The topological phase transitions in the 2D crystals can be understood based on intrinsic spin orbit coupling which arises due to perpendicular electric field or interaction with a substrate. Kane and Mele^21^ applied a model Hamiltonian to describe topological insulators. Such model consists of a hopping and an intrinsic spin-orbit term on the Graphene like structures. The Kane-Mele model essentially includes two copies with different sign for up and down spins of a model introduced earlier by Haldane^22^. Such microscopic model was originally proposed to describe the quantum spin Hall effect in Graphene^21^. Subsequent band structure calculations showed, however, that the spin orbit gap in Graphene is so small^23,24^ that the quantum spin Hall effect in Graphene like structures is beyond experimental relevance.

In-plane magnetic field affects the magneto conductivity of honeycomb structures so that the results show the negative for intrinsic gapless Graphene. However the magneto-resistance of gapless Graphene presents a positive value for fields lower than the critical magnetic field and negative above the critical magnetic^25^. Moreover, microwave magneto transport in doped Graphene is an open problem^26^.

The many body effects such as Coulomb interaction and its dynamical screening present the novel features for any electronic material. The collective spectrum and quasiparticle properties of electronic systems are determined form dynamical spin structure factors. These are applied to imply the optical properties of the system. Moreover a lot of studies have been done on collective modes of monolayer Graphene both theoretically^27,28^ and experimentally^29^. However there are no the extensive theoretical studies on doped bilayer systems.

The frequency dependence of dynamical spin susceptibility has been studied and the results causes to find the collective magnetic excitation spectrum of many body system. It is worthwhile to explain the experimental interpretation of imaginary part of dynamical spin susceptibilities. Slow neutrons scatter from solids via magnetic dipole interaction in which the magnetic moment of the neutron interacts with the spin magnetic moment of electrons in the solid^30^. We can readily express the inelastic cross-section of scattering of neutron beam from a magnetic system based on correlation functions between spin density operators. In other words the differential inelastic cross section $d^{2}\sigma /d\Omega d\omega$ corresponds to imaginary part of spin susceptibilities. $\omega$ describes the energy loss of neutron beam which is defined as the difference between incident and scattered neutron energies. $\Omega$ introduces solid angle of scattered neutrons. The spin excitation modes of the magnetic system have been found via the frequency position of peaks in $d^{2}\sigma /d\Omega d\omega$. Depending on component of spin magnetic moment of electrons that interacts with spin of neutrons, transverse and longitudinal spin susceptibility behaviors have been investigated. The imaginary and real part of non-interacting change susceptibilities of Graphene within an analytical approach have been calculated A theoretical work has been performed for calculating both^31^. The results of this study shows there is no remarkable angle dependence for imaginary part of polarizability around the van Hove singularity, i.e, $\hbar \omega /t=2.0$ where *t* implies nearest neighbor hopping integral.

It is worthwhile to add few comments regarding the comparison Germanene and Graphene like structure. As we have mentioned, the most important difference between Germanene structure ad Graphene one arises from the nonzero overlap function between nearest neighbor atoms in Germanene structure. Response functions of Graphene in the presence of spin-orbit coupling have been studied recently^32,33^. In this references, the optical absorption of Graphene structure in the presence of spin-orbit coupling and magnetic field has been theoretically studied. Optical absorption rate corresponds to the charge transition rate of electrons between energy levels. However in our work, we have investigated the dynamical spin susceptibility of Germanene structure due to spin-orbit coupling. In other words we have specially studied the transition rate of magnetic degrees freedom of electrons. Such study is a novelty of our work so that there is no the theoretical work on the study of magnetic excitation modes in Germanene structure due to spin-orbit coupling. These studies have been performed for Graphene like structures and the most important difference between our results and the spin susceptibilities of Graphene structure is the effects of overlap function in Germanene structure compared to Graphene lattice. In fact overlap function has considerable impact on the frequency position of excitation mode and also on the intensity of scattered neutron beam from Germanene structure.

The purpose of this paper is to provide a Kane Mele model including intrinsic spin-orbit interaction for studying frequency behavior of dynamical spin susceptibility of Germanene layer in the presence of magnetic field perpendicular to the plane. Using the suitable hopping integral and on site parameter values, the band dispersion of electrons has been calculated. Full band calculation beyond Dirac approximation has been implemented to derive both transverse and longitudinal dynamical spin susceptibilities. We have exploited Green’s function approach to calculate the spin susceptibility, i.e. the time ordered spin operator correlation. The effects of electron doping, magnetic field and spin-orbit coupling on the spin structure factors have been studied. Also we discuss and analyze to show how spin-orbit coupling affects the frequency behavior of the longitudinal and transverse spin susceptibilities. Also we study the frequency behavior of dynamical spin susceptibility of Germanene due to variation of chemical potential and magnetic field. Also the effects of spin-orbit coupling constant and magnetic field on temperature dependence of both transverse and longitudinal static spin susceptibilities have been investigated in details.

## Model Hamiltonian and formalism

**Figure 1:**
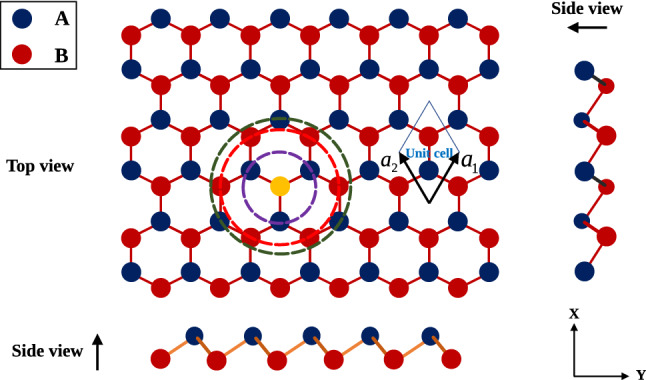
Crystal structure of Germanene.

**Figure 2:**
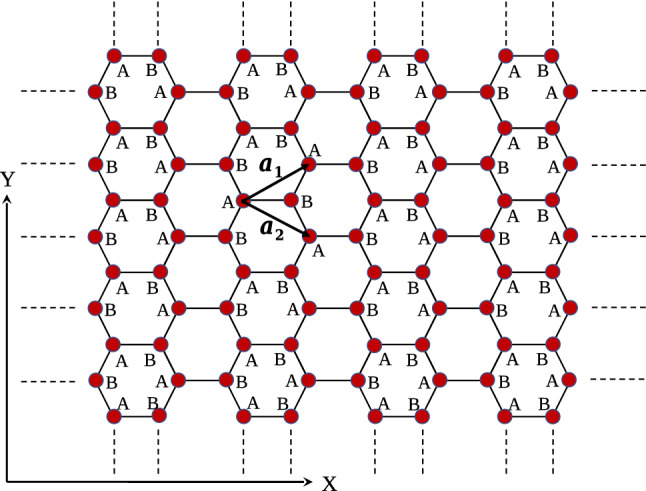
The structure of honeycomb structure is shown. The light dashed lines denote the Bravais lattice unit cell. Each cell includes two nonequivalent sites, which are indicated by A and B. $\mathbf{a}_{1}$ and $\mathbf{a}_{2}$ are the primitive vectors of unit cell.

The crystal structure of Germanene has been shown in Fig. 1. The unit cell of Germanene structure is similar to Graphene layer and this honeycomb lattice depicted in Fig. 2. The primitive unit cell vectors of honeycomb lattice have been shown by $\mathbf{a}_{1}$ and $\mathbf{a}_{2}$. In the presence of longitudinal magnetic field, the Kane-Mele model^21^ (*H*) for Germanene structure includes the tight binding model ($H^{TB}$), the intrinsic spin-orbit coupling ($H^{ISOC}$) and the Zeeman term ($H^{Zeeman}$) due to the coupling of spin degrees of freedom of electrons with external longitudinal magnetic field *B*

1 $$\begin{aligned} H= & {} H^{TB}+H^{ISOC}+H^{Zeeman}. \end{aligned}$$

The tight binding part of model Hamiltonian consists of three parts; nearest neighbor hopping, next nearest neighbor (2NN) hopping and next next nearest neighbor (3NN) hopping terms. The tight binding part, the spin orbit coupling term and the Zeeman part of the model Hamiltonian on the honeycomb lattice are given by

2 $$\begin{aligned} H^{TB}= & {} -t\sum _{i,{\varvec{\Delta }},\sigma }\Big (a^{\sigma \dag }_{j=i+{\varvec{\Delta }}}b^{\sigma }_{i}+h.c.\Big ) -t^{\prime }\sum _{i,{\varvec{\Delta }}^{\prime },\sigma }\Big (a^{\sigma \dag }_{j=i+{\varvec{\Delta }}^{\prime }}a^{\sigma }_{i}+b^{\sigma \dag }_{i+{\varvec{\Delta }}^{\prime }}b^{\sigma }_{i}\Big ) -t^{\prime \prime }\sum _{i,{\varvec{\Delta }}^{\prime \prime },\sigma }\Big (a^{\sigma \dag }_{j=i+{\varvec{\Delta }}^{\prime \prime }}b^{\sigma }_{i}+h.c.\Big ) \nonumber \\&-\sum _{i,\sigma } \mu \Big (a^{\dag \sigma }_{i}a^{\sigma }_{i}+b^{\dag \sigma }_{i} b^{\sigma }_{i}\Big ),\nonumber \\ H^{ISOC}= & {} i\lambda \sum _{i,{\varvec{\Delta }}^{\prime },\sigma }\sum _{\alpha =A,B}\Big (\nu ^{a}_{i+\Delta ^{\prime },i} a^{\dag \sigma }_{i+{\varvec{\Delta }}^{\prime }}\sigma ^{z}_{\sigma \sigma ^{\prime }} a^{\sigma ^{\prime }}_{i}+\nu ^{b}_{i+{\varvec{\Delta }}^{\prime },i} b^{\dag \sigma }_{i+{\varvec{\Delta }}^{\prime }}\sigma ^{z}_{\sigma \sigma ^{\prime }} b^{\sigma ^{\prime }}_{i}\Big ),\nonumber \\ H^{Zeeman}= & {} -\sum _{i,\sigma }\sigma g\mu _{B}B\Big (a^{\dag \sigma }_{i}a^{\sigma }_{i}+b^{\dag \sigma }_{i} b^{\sigma }_{i}\Big ). \end{aligned}$$

Here $a^{\sigma }_{i}(b^{\sigma }_{i})$ is an annihilation operator of electron with spin $\sigma$ on sublattice *A*(*B*) in unit cell index *i*. The operators fulfill the fermionic standard anti commutation relations $\{a^{\sigma }_{i},a^{\sigma ^{\prime }\dag }_{j}\}= \delta _{ij}\delta _{\sigma \sigma ^{\prime }}$. As usual $t,t^{\prime },t^{\prime \prime }$ denote the nearest neighbor, next nearest neighbor and next next nearest neighbor hopping integral amplitudes, respectively. The parameter $\lambda$ introduces the spin-orbit coupling strength. Also *B* refers to strength of applied magnetic field. *g* and $\mu _{B}$ introduce the gyromagnetic and Bohr magneton constants, respectively. $\sigma ^{z}$ is the third Pauli matrix, and $\nu _{ji}^{a(b)}=\pm 1$ as discussed below. Based on Fig. 2, $\mathbf{a}_{1}$ and $\mathbf{a}_{2}$ are the primitive vectors of unit cell and the length of them is assumed to be unit. The symbol ${\varvec{\Delta }}=\mathbf{0},{\varvec{\Delta }}_{1},{\varvec{\Delta }}_{2}$ implies the indexes of lattice vectors connecting the unit cells including nearest neighbor lattice sites. The translational vectors ${\varvec{\Delta }}_{1},{\varvec{\Delta }}_{2}$ connecting neighbor unit cells are given by

3 $$\begin{aligned} {\varvec{\Delta }}_{1}=\mathbf{i}\frac{\sqrt{3}}{2}+\mathbf{j}\frac{1}{2}\;\;,\;\;{\varvec{\Delta }}_{2}= \mathbf{i}\frac{\sqrt{3}}{2}-\mathbf{j}\frac{1}{2}. \end{aligned}$$

Also index ${\varvec{\Delta }}^{\prime }={\varvec{\Delta }}_{1},{\varvec{\Delta }}_{2}, -{\varvec{\Delta }}_{1},-{\varvec{\Delta }}_{2},\mathbf{j},-\mathbf{j}$ implies the characters of lattice vectors connecting the unit cells including next nearest neighbor lattice sites. Moreover index ${\varvec{\Delta }}^{\prime \prime }=\sqrt{3}{} \mathbf{i},\mathbf{j},-\mathbf{j}$ denotes the characters of lattice vectors connecting the unit cells including next next nearest neighbor lattice sites. We consider the intrinsic spin-orbit term^21^ of the KM Hamiltonian in Eq. (2). The expression $\nu _{ji}^{a(b)}$ gives $\pm 1$ depending on the orientation of the sites. A standard definition for $\nu ^{\alpha }_{ji}$ in each sublattice $\alpha =A,B$ is $\nu _{ji}^{\alpha }=\Big ( \frac{\mathbf{d}^{\alpha }_{j}\times \mathbf{d}^{\alpha } _{i}}{|\mathbf{d}^{\alpha }_{j}\times \mathbf{d}^{\alpha }_{i}|}\Big ).\mathbf{e}_{z}=\pm 1$ where $\mathbf{d} ^{\alpha }_{j}$ and $\mathbf{d} ^{\alpha }_{i}$ are the two unit vectors along the nearest neighbor bonds connecting site *i* to its next-nearest neighbor *j*. Moreover $\mathbf{e}_{z}$ implies the unit vector perpendicular to the plane. Because of two sublattice atoms, the band wave function $\psi ^{\sigma }_{n}(\mathbf{k},\mathbf{r})$ can be expanded in terms of Bloch functions $\Phi ^{\sigma }_{\alpha }(\mathbf{k},\mathbf{r})$. The index $\alpha$ implies two inequivalent sublattice atoms *A*, *B* in the unit cell, $\mathbf{r}$ denotes the position vector of electron, $\mathbf{k}$ is the wave function belonging in the first Brillouin zone of honeycomb structure. Such band wave function can be written as

4 $$\begin{aligned} \psi ^{\sigma }_{n}(\mathbf{k},\mathbf{r})=\sum _{\alpha =A,B}C^{\sigma }_{n\alpha }(\mathbf{k}) \Phi ^{\sigma }_{\alpha }(\mathbf{k},\mathbf{r}), \end{aligned}$$

where $C^{\sigma }_{n\alpha }(\mathbf{k})$ is the expansion coefficients and $n=c,v$ refers to condition and valence bands. Also we expand the Bloch wave function in terms of Wannier wave function as

5 $$\begin{aligned} \Phi ^{\sigma }_{\alpha }(\mathbf{k},\mathbf{r})=\frac{1}{\sqrt{N}}\sum _{\mathbf{R}_{i}} e^{i\mathbf{k}.\mathbf{R}_{i}}\phi ^{\sigma }_{\alpha }(\mathbf{r}-\mathbf{R}_{i}), \end{aligned}$$

so that $\mathbf{R}_{i}$ implies the position vector of *i*th unit cell in the crystal and $\phi _{\alpha }$ is the Wannier wave function of electron in the vicinity of atom in *i* th unit cell on sublattice index $\alpha$. By inverting the expansion Eq. (4), we can expand the Bloch wave functions in terms of band wave function as following relation

6 $$\begin{aligned} \Phi ^{\sigma }_{\alpha }(\mathbf{k},\mathbf{r})=\sum _{n=c,v}D_{\alpha n}^{\sigma }(\mathbf{k}) \psi ^{\sigma }_{n}(\mathbf{k},\mathbf{r}), \end{aligned}$$

where $D_{\alpha n}^{\sigma }(\mathbf{k})$ is the expansion coefficients and we explain these coefficients in the following.

The small Buckling in Germanene causes to the considerable value for 2NN and 3NN hopping amplitude. Moreover we have considerable values for overlap parameters of electron wave functions between 2NN and 3NN atoms. The band structures of electrons with spin $\sigma$ of Germanene described by model Hamiltonian in Eq. (2) are obtained by using the matrix form of Schrodinger as follows

7 $$\begin{aligned} {{\mathcal {H}}}^{\sigma }(\mathbf{k}){{\mathcal {C}}}^{\sigma }(\mathbf{k})= & {} E^{\sigma }_{n}(\mathbf{k}){{\mathcal {S}}}^{\sigma } (\mathbf{k}){{\mathcal {C}}}^{\sigma }(\mathbf{k}),\nonumber \\ {{\mathcal {H}}}^{\sigma }(\mathbf{k})= & {} \left( \begin{array}{cc} H^{\sigma }_{AA}(\mathbf{k})&{} H^{\sigma }_{AB}(\mathbf{k}) \\ H_{BA}^{\sigma }(\mathbf{k}) &{} H^{\sigma }_{BB}(\mathbf{k}) \\ \end{array} \right) \;,\;{{\mathcal {C}}}^{\sigma }(\mathbf{k})=\left( \begin{array}{c} C_{nA}^{\sigma }(\mathbf{k})\\ C_{nB}^{\sigma }(\mathbf{k})\\ \end{array} \right) ,\;\;\nonumber \\ {{\mathcal {S}}}^{\sigma }(\mathbf{k})= & {} \left( \begin{array}{cc} S^{\sigma }_{AA}(\mathbf{k})&{} S^{\sigma }_{AB}(\mathbf{k}) \\ S^{\sigma }_{BA}(\mathbf{k}) &{} S^{\sigma }_{BB}(\mathbf{k})\\ \end{array} \right) . \end{aligned}$$

Using the Bloch wave functions, i.e. $\Phi _{\alpha }(\mathbf{k})$, the matrix elements of ${{\mathcal {H}}}$ and ${{\mathcal {S}}}$ are given by

8 $$\begin{aligned} H^{\sigma }_{\alpha \beta }(\mathbf{k})=\langle \Phi ^{\sigma }_{\alpha }(\mathbf{k})|H|\Phi ^{\sigma }_{\beta }(\mathbf{k})\rangle \;\;,\;\; S^{\sigma }_{\alpha \beta }(\mathbf{k})=\langle \Phi ^{\sigma }_{\alpha }(\mathbf{k})|\Phi ^{\sigma }_{\beta }(\mathbf{k})\rangle . \end{aligned}$$

The matrix elements of $H^{\sigma }_{\alpha \beta }$ and $S^{\sigma }_{\alpha \beta }$ are expressed based on hopping amplitude and spin-orbit coupling between two neighbor atoms on lattice sites and can be expanded in terms of hopping amplitudes $t,t^{\prime },t^{\prime \prime }$, spin orbit coupling $\lambda$ and overlap parameters. The diagonal elements of matrixes ${{\mathcal {H}}}$ in Eq. (7) arise from hopping amplitude of electrons between next nearest neighbor atoms on the same sublattice and spin-orbit coupling. Also the off diagonal matrix elements with spin channel $\sigma$, i.e. $H^{\sigma }_{AB}, H^{\sigma }_{BA}$, raise from hopping amplitude of electrons between nearest neighbor atoms and next next nearest neighbor atoms on the different sublattices. These matrix elements are obtained as

9 $$\begin{aligned} H^{\sigma }_{AB}(\mathbf{k})= & {} t\left( 1+e^{i\mathbf{k}.{\varvec{\Delta }}_{1}}+e^{i\mathbf{k}.{\varvec{\Delta }}_{2}}\right) +t^{\prime \prime } \left( 2\cos (k_{y})+e^{-i\sqrt{3}k_{x}}\right) \nonumber \\= & {} t\left( 1+2\cos (k_{y}/2)e^{-i\sqrt{3}k_{x}/2}\right) +t^{\prime \prime } \left( 2\cos (k_{y})+e^{-i\sqrt{3}k_{x}}\right) ,\nonumber \\ H^{\sigma }_{AA}(\mathbf{k})= & {} 2t^{\prime }\left( \cos \left( \sqrt{3}k_{x}/2+k_{y}/2\right) +\cos \left( \sqrt{3}k_{x}/2+k_{y}/2\right) +\cos (k_{y}/2)\right) \nonumber \\&-2\lambda \left( \sin \left( \frac{1}{2}k_{y}\right) - \sin \left( \frac{\sqrt{3}}{2}k_{x}+\frac{1}{2}k_{y}\right) - \sin \left( \frac{\sqrt{3}}{2}k_{x}-\frac{1}{2}k_{y}\right) \right) -\mu -\sigma g\mu _{B}B,\nonumber \\ H^{\sigma }_{BB}(\mathbf{k})= & {} -2t^{\prime }\left( \cos \left( \sqrt{3}k_{x}/2+k_{y}/2\right) +\cos \left( \sqrt{3}k_{x}/2+k_{y}/2\right) +\cos (k_{y}/2)\right) \nonumber \\&+2\lambda \left( \sin \left( \frac{1}{2}k_{y}\right) - \sin \left( \frac{\sqrt{3}}{2}k_{x}+\frac{1}{2}k_{y}\right) - \sin \left( \frac{\sqrt{3}}{2}k_{x}-\frac{1}{2}k_{y}\right) \right) -\mu -\sigma g\mu _{B}B,\nonumber \\ H^{\sigma }_{BA}(\mathbf{k})= & {} H^{*}_{AB}(\mathbf{k}). \end{aligned}$$

Based on matrix elements $H^{\sigma }_{\alpha \beta }(\mathbf{k})$, the model Hamiltonian in Eq. (2) is written in terms of Fourier transformation of creation and annihilation fermionic operators as

10 $$\begin{aligned} H=\sum _{\mathbf{k},\sigma }\left[ H^{\sigma }_{AA}(\mathbf{k})a^{\sigma \dag }_\mathbf{k}a^{\sigma }_\mathbf{k} +H_{AB}(\mathbf{k})a^{\sigma \dag }_\mathbf{k}b^{\sigma }_\mathbf{k}+ H_{BA}(\mathbf{k})b^{\sigma \dag }_\mathbf{k}a^{\sigma }_\mathbf{k}+ H^{\sigma }_{BB}(\mathbf{k})b^{\sigma \dag }_\mathbf{k}b^{\sigma }_\mathbf{k}\right] , \end{aligned}$$

so that the operator $a^{\sigma }_\mathbf{k}(b^{\sigma }_\mathbf{k})$ annihilates an electron at wave vector $\mathbf{k}$ with spin index $\sigma$ on sublattice A(B) and has the following relation as

11 $$\begin{aligned} a^{\sigma }_\mathbf{k}=\frac{1}{\sqrt{N}}\sum _\mathbf{k}e^{i\mathbf{k}\cdot \mathbf{R}_{i}}a^{\sigma }_{i} \;\;,\;\;b^{\sigma }_\mathbf{k}=\frac{1}{\sqrt{N}}\sum _\mathbf{k}e^{i\mathbf{k}\cdot \mathbf{R}_{i}}b^{\sigma }_{i}. \end{aligned}$$

The matrix elements of ${{\mathcal {S}}}(\mathbf{k})$, i.e. $S_{AA}(\mathbf{k})$ , $S_{AB}(\mathbf{k})$, $S_{BA}(\mathbf{k})$ and $S_{BB}(\mathbf{k})$ are expressed as

12 $$\begin{aligned} S_{AB}(\mathbf{k})= & {} s\Big (1+e^{i\mathbf{k}.{\varvec{\Delta }}_{1}}+e^{i\mathbf{k}.{\varvec{\Delta }}_{2}}\Big )+s^{\prime \prime } \Big (2\cos (k_{y})+e^{-i\sqrt{3}k_{x}}\Big )\nonumber \\= & {} s\Big (1+2\cos (k_{y}/2)e^{-i\sqrt{3}k_{x}/2}\Big )+s^{\prime \prime } \Big (2\cos (k_{y})+e^{-i\sqrt{3}k_{x}}\Big )\nonumber \\ S_{AA}(\mathbf{k})= & {} 1+2s^{\prime }\Big (\cos \left( \sqrt{3}k_{x}/2+k_{y}/2\right) +\cos \left( \sqrt{3}k_{x}/2+k_{y}/2\right) +\cos (k_{y}/2)\Big )\nonumber \\ S_{BB}(\mathbf{k})= & {} S_{AA}(\mathbf{k})\;\;,\;\;S_{BA}(\mathbf{k})=S^{*}_{AB}(\mathbf{k}), \end{aligned}$$

so that *s* is the overlap between orbital wave function of electron respect to the nearest neighbor atoms, $s^{\prime }$ denotes the overlap between orbital wave function of electron respect to the next nearest neighbor atoms and $s^{\prime \prime }$ implies the overlap between orbital wave function of electron respect to the next next nearest neighbor atoms. The density functional theory and *ab initio* calculations has been determined the hopping amplitudes and overlap values $s,s^{\prime },s^{\prime \prime }$ as^18^ $t=-1.163,t^{\prime }=-0.055,t^{\prime \prime }=-0.0836,s=0.01207,s^{\prime }=0.0128,s^{\prime \prime }=0.048$. Using the Hamiltonian and overlap matrix forms in Eqs. (9, 12), the band structure of electrons, i.e. $E^{\sigma }_{\eta }(\mathbf{k})$ has been found by solving equation $\mathrm{\det }\Big ({{\mathcal {H}}}(\mathbf{k})-E(\mathbf{k}){{\mathcal {S}}}(\mathbf{k})\Big )=0$. Moreover the matrix elements of ${\mathcal {C}}(\mathbf{k})$ can be found based on eigenvalue equation in Eq. (7). Equation (7) can be rewritten as matrix equation as follows

13 $$\begin{aligned} \left( \begin{array}{cc} \psi _{c}(\mathbf{k},\mathbf{r}) \\ \psi _{v}(\mathbf{k},\mathbf{r}) \\ \end{array} \right) =\left( \begin{array}{cc} C^{\sigma }_{cA}(\mathbf{k})&{} C^{\sigma }_{cB}(\mathbf{k}) \\ C^{\sigma }_{vA}(\mathbf{k}) &{} C^{\sigma }_{vB}(\mathbf{k})\\ \end{array} \right) \left( \begin{array}{c} \Phi ^{\sigma }_{A}(\mathbf{k},\mathbf{r})\\ \Phi ^{\sigma }_{B}(\mathbf{k},\mathbf{r})\\ \end{array} \right) . \end{aligned}$$

In a similar way, we can express the matrix from for Eq. (6)

14 $$\begin{aligned} \left( \begin{array}{cc} \Phi ^{\sigma }_{A}(\mathbf{k},\mathbf{r}) \\ \Phi ^{\sigma }_{B}(\mathbf{k},\mathbf{r}) \\ \end{array} \right) =\left( \begin{array}{cc} D^{\sigma }_{Ac}(\mathbf{k})&{} D^{\sigma }_{Av}(\mathbf{k}) \\ D^{\sigma }_{Bc}(\mathbf{k}) &{} D^{\sigma }_{Bv}(\mathbf{k})\\ \end{array} \right) \left( \begin{array}{c} \psi ^{\sigma }_{c}(\mathbf{k},\mathbf{r})\\ \psi ^{\sigma }_{v}(\mathbf{k},\mathbf{r})\\ \end{array} \right) ,\nonumber \\ \left( \begin{array}{cc} D^{\sigma }_{Ac}(\mathbf{k})&{} D^{\sigma }_{Av}(\mathbf{k}) \\ D^{\sigma }_{Bc}(\mathbf{k}) &{} D^{\sigma }_{Bv}(\mathbf{k})\\ \end{array} \right) =\left( \begin{array}{cc} C^{\sigma }_{cA}(\mathbf{k})&{} C^{\sigma }_{cB}(\mathbf{k}) \\ C^{\sigma }_{vA}(\mathbf{k}) &{} C^{\sigma }_{vB}(\mathbf{k})\\ \end{array} \right) ^{-1} \end{aligned}$$

The final results for band structure and expansion coefficients, i.e. $C^{\sigma }_{n\alpha }$ and $D^{\sigma }_{\alpha n}$, are lengthy and are not given here. The valence and condition bands of electrons have been presented by $E^{\sigma }_{v}(\mathbf{k})$ and $E^{\sigma }_{c}(\mathbf{k})$ respectively. In the second quantization representation, we can rewrite the Eq. (14) as

15 $$\begin{aligned} \left( \begin{array}{cc} a^{\sigma \dag }_{\mathbf{k}} \\ b^{\sigma \dag }_{\mathbf{k}} \\ \end{array} \right) =\left( \begin{array}{cc} D^{\sigma }_{Ac}(\mathbf{k})&{} D^{\sigma }_{Av}(\mathbf{k}) \\ D^{\sigma }_{Bc}(\mathbf{k}) &{} D^{\sigma }_{Bv}(\mathbf{k})\\ \end{array} \right) \left( \begin{array}{c} c^{\sigma \dag }_{c,\mathbf{k}}\\ c^{\sigma \dag }_{v,\mathbf{k}}\\ \end{array} \right) , \end{aligned}$$

Using band energy spectrum, the Hamiltonian in Eq. (2) can be rewritten by

16 $$\begin{aligned} H=\sum _{\mathbf{k},\sigma ,\eta =c,v} E^{\sigma }_{\eta }(\mathbf{k})c^{\sigma \dag }_{\eta ,\mathbf{k}}c^{\sigma }_{\eta ,\mathbf{k}}, \end{aligned}$$

where $c^{\sigma }_{\eta ,\mathbf{k}}$ defines the creation operator of electron with spin $\sigma$ in band index $\eta$ at wave vector $\mathbf{k}$. Since longitudinal magnetic field has been applied perpendicular to the Germanene layer, the electronic Green’s function depends on the spin index $\sigma =\uparrow ,\downarrow$. According to the model Hamiltonian introduced in Eq. (2), the elements of spin resolved Matsubara Green’s function are introduced as the following forms

17 $$\begin{aligned} G^{\sigma }_{AA}(\mathbf{k},\tau )= & {} -\langle \mathcal{T}(a_{\mathbf{k},\sigma }(\tau )a^{\dag }_{\mathbf{k},\sigma }(0))\rangle \;\;,\;\; G^{\sigma }_{AB}(\mathbf{k},\tau )=-\langle \mathcal{T}(a_{\mathbf{k},\sigma }(\tau )b^{\dag }_{\mathbf{k},\sigma }(0))\rangle ,\nonumber \\ G^{\sigma }_{BA}(\mathbf{k},\tau )= & {} -\langle \mathcal{T}(b_{\mathbf{k},\sigma }(\tau )a^{\dag }_{\mathbf{k},\sigma }(0))\rangle \;\;,\;\; G^{\sigma }_{BB}(\mathbf{k},\tau )=-\langle \mathcal{T}(b_{\mathbf{k},\sigma }(\tau )b^{\dag }_{\mathbf{k},\sigma }(0))\rangle . \end{aligned}$$

*T* introduces the time ordering operator and arranges the creation and annihilation operators in terms of time of them without attention to the their algebra. The Fourier transformation of each Green’s function element is obtained by

18 $$\begin{aligned} G^{\sigma }_{\alpha \beta }(\mathbf{k},i\omega _{m})=\int ^{1/k_{B}T}_{0} d\tau e^{i\omega _{m}\tau }G^{\sigma }_{\alpha \beta }(\mathbf{k},\tau )\;\;,\;\;\alpha ,\beta =A,B, \end{aligned}$$

where $\omega _{m}=(2m+1)\pi k_{B}T$ denotes the Fermionic Matsubara frequency. After some algebraic calculation, the following expression is obtained for Green’s functions in Fourier presentation

19 $$\begin{aligned} G^{\sigma }_{\alpha \beta }(\mathbf{k},i\omega _{m})= & {} \sum _{\eta =c,v}\frac{D_{\alpha \eta }^{\sigma *}(\mathbf{k}) D^{\sigma }_{\beta \eta }(\mathbf{k})}{i\omega _{m}-E^{\sigma }_{\eta }(\mathbf{k})}, \end{aligned}$$

where $\alpha ,\beta$ refer to the each atomic basis of honeycomb lattice and $E^{\sigma }_{\eta }(\mathbf{k})$ is the band structure of Germanene layer in the presence of magnetic field and spin-orbit coupling. For determining the chemical potential, $\mu _{\sigma }$, we use the relation between concentration of electrons ($n_{e}$) and chemical potential. This relation is given by

20 $$\begin{aligned} n_{e}=\frac{1}{4N}\sum _{\mathbf{k},\eta ,\sigma }\frac{1}{e^{E^{\sigma }_{\eta }(\mathbf{k})/k_{B}T}+1}. \end{aligned}$$

In fact the diagonal matrix elements of the Hamiltonian in Eq. (9) depends on chemical potential $\mu$. Thus eigenvalues, i.e. $E^{\sigma }_{\eta }(\mathbf{k})$ includes the factor $\mu$. Therefore the right hand of Eq. (20) depends on chemical potential $\mu$. With an initial guess for chemical potential $\mu$, we can solve the algebraic eq. (20) so that we can find the chemical potential value for each amount for electronic concentration $n_{e}$. These statements have been added to the manuscript after Eq. (20). Based on the values of electronic concentration $n_{e}$, the chemical potential, $\mu$, can be obtained by means Eq. (20). In order to obtain the magnetic excitation spectrum of Germanene structure both transverse and longitudinal dynamical spin susceptibilities have been presented using Green’s function method in the following section.

## Dynamical and static spin structure factors

The correlation function between spin components of itinerant electrons in Germanene layer at different times can be expressed in terms of one particle Green’s functions. The frequency Fourier transformation of this correlation function produces the dynamical spin susceptibility. The frequency position of peaks in dynamical spin susceptibility are associated with collective excitation of electronic gas described by Kane-Mele model Hamiltonian in the presence of magnetic field. These excitations are related to the spin excitation spectrum of electrons on Honeycomb structure. In the view point of experimental interpretation, the dynamical spin susceptibility of the localized electrons of the system is proportional to inelastic cross-section for magnetic neutron scattering from a magnetic system that can be expressed in terms of spin density correlation functions of the system. In other words the differential inelastic cross section $d^{2}\sigma /d\Omega d\omega$ is proportional to imaginary part of spin susceptibilities. $\omega$ denotes the energy loss of neutron beam which is defined as the difference between incident and scattered neutron energies. The solid angle $\Omega$ implies the orientation of wave vector of scattered neutrons from the localized electrons of the sample. We can assume the wave vector of incident neutrons is along *z* direction. The solid angle $\Omega$ depends on the polar angle between wave vector of scattered neutrons and the wave vector of the incident neutrons. The frequency position of peaks in $d^{2}\sigma /d\Omega d\omega$ determines the spin excitation spectrum of the magnetic system^30^. In order to study the general spin excitation spectrum of the localized electron of the systems, both transverse and longitudinal dynamical spin-spin correlation functions have been calculated. Linear response theory gives us the dynamical spin response functions based on the correlation function between components of spin operators. We introduce $\chi _{+-}$ as transverse spin susceptibility and its relation is given by

21 $$\begin{aligned} \chi _{+-}(\mathbf{q},\omega )= & {} i \int _{-\infty }^{+\infty }dt e^{i\omega t} \langle [S^{+}(\mathbf{q},t),S^{-}(-\mathbf{q},0)]\rangle \nonumber \\= & {} \lim _{i\Omega _{n} \longrightarrow \omega +i0^{+}}\int _{0}^{1/(k_{B}T)}d\tau e^{i\Omega _{n}\tau }\langle {\mathcal {T}}S^{+}(\mathbf{q},\tau )S^{-}(-\mathbf{q},0)\rangle \nonumber \\= & {} \chi _{+-}(\mathbf{q},i\Omega _{n}\longrightarrow \omega +i0^{+}), \end{aligned}$$

in which $\Omega _{n}=2n\pi k_{B}T$ is the bosonic Matsubara frequency and ${\mathcal {T}}$ implies the time order operator. Also the wave vector $\mathbf{q}$ in Eq. (21) implies the difference between incident and scattered neutron wave vectors. The Fourier transformations of transverse components of spin density operators, ($S^{+(-)}$), in terms of fermionic operators is given by

22 $$\begin{aligned} S^{+}(\mathbf{q})= & {} \sum _\mathbf{k}\Big (a^{\dag \uparrow }_{\mathbf{k}+\mathbf{q}} a^{\downarrow }_\mathbf{k}+b^{\dag \uparrow }_{\mathbf{k}+\mathbf{q}} b^{\downarrow }_\mathbf{k}\Big )\;\;,\;\; S^{-}(\mathbf{q})=\sum _\mathbf{k}\Big (a^{\dag \downarrow }_{\mathbf{k}+\mathbf{q}} a^{\uparrow }_\mathbf{k}+b^{\dag \downarrow }_{\mathbf{k}+\mathbf{q}} b^{\uparrow }_\mathbf{k}\Big ). \end{aligned}$$

Substituting the operator form of $S^{+}$ and $S^{-}$ into definition of transverse spin susceptibility in Eq. (21), we arrive the following expression for transverse dynamical spin susceptibility ($\chi _{+-}(\mathbf{q},i\Omega _{n})$)

23 $$\begin{aligned} \chi _{+-}(\mathbf{q},i\Omega _{n})= & {} \int _{0}^{1/(k_{B}T)}d\tau e^{i\Omega _{n}\tau }\frac{1}{N^{2}} \sum _{\mathbf{k},\mathbf{k}^{\prime }}\Big \langle \mathcal{T}\Big ( a^{\dag \uparrow }_{\mathbf{k}+\mathbf{q}}(\tau ) a^{\downarrow }_\mathbf{k}(\tau )+b^{\dag \uparrow }_{\mathbf{k}+\mathbf{q}}(\tau ) b^{\downarrow }_\mathbf{k}(\tau )\Big )\nonumber \\&\times \Big (a^{\dag \downarrow }_{\mathbf{k}+\mathbf{q}}(0) a^{\uparrow }_\mathbf{k}(0)+b^{\dag \downarrow }_{\mathbf{k}+\mathbf{q}}(0) b^{\uparrow }_\mathbf{k}(0)\Big ) \Big \rangle , \end{aligned}$$

that *N* is the number of unit cells in Germanene structure. In order to calculate the correlation function in Eq. (23), one particle spin dependent Green’s function matrix elements presented in Eq. (19) should be exploited. After applying Wick’s theorem and taking Fourier transformation, we can transverse susceptibility in terms of one particle spin dependent Green’s function

24 $$\begin{aligned} \chi _{+-}(\mathbf{q},i\Omega _{n})=- \frac{k_{B}T}{N}\sum _{\mathbf{k}}\sum _{\alpha ,\beta }\sum _{m} G^{\uparrow }_{\beta \alpha }(\mathbf{k},i\omega _{m})G^{\downarrow }_ {\alpha \beta }(\mathbf{k}+\mathbf{q},i\Omega _{n}+i\omega _{m}). \end{aligned}$$

The applied magnetic field to the Germanene layer causes to the spin dependent property for one particle Green’s function. In order to perform summation over Matsubara frequency $\omega _{m}$ in Eq. (24), the Matsubara Green’s function elements should be written in terms of imaginary part of retarded Green’s function matrix elements using Lehman equation^34^ as

25 $$\begin{aligned} G^{\sigma }_{\alpha \beta }(\mathbf{k},i\omega _{m})=\int ^{+\infty }_{-\infty }\frac{d\epsilon }{2\pi }\frac{-2{\mathrm{ImG}}^{\sigma }_{\alpha \beta }(\mathbf{k},i\omega _{m}\longrightarrow \epsilon +i0^{+})}{i\omega _{m}-\epsilon }, \end{aligned}$$

where $G^{\sigma }_{\alpha \beta }(\mathbf{k},i\omega _{m}\longrightarrow \epsilon +i0^{+})$ denotes the retarded Green’s function matrix element. By replacing Lehman representation for Matsubara Green’s function matrix elements into Eq. (24) and taking summation over fermionic Matsubara frequency $\omega _{m}$, we obtain dynamical transverse spin susceptibility of electrons on Germanene structure as following form

26 $$\begin{aligned} \chi _{+-}(\mathbf{q},i\Omega _{n})= & {} - \frac{1}{N}\sum _{\mathbf{k}}\sum _{\alpha ,\beta }\int ^{+\infty }_{-\infty }\int ^{+\infty } _{-\infty }\frac{d\epsilon d\epsilon ^{\prime }}{\pi ^{2}} {\mathrm{ImG}} ^{\uparrow }_{\beta \alpha }(\mathbf{k},\epsilon +i0^{+}){\mathrm{ImG}} ^{\downarrow }_{\alpha \beta }(\mathbf{k}+\mathbf{q},\epsilon ^{\prime }+i0^{+})\nonumber \\&\times \frac{n_{F}(\epsilon )-n_{F}(\epsilon ^{\prime })}{i\Omega _{n}+\epsilon -\epsilon ^{\prime }}, \end{aligned}$$

where $n_{F}(x)=\frac{1}{e^{x/k_{B}T}+1}$ implies well known Fermi-Dirac distortion function. The Fourier transformation of longitudinal component of the spin, i.e. $S^{z}(\mathbf{q})$, is given in terms of fermionic operators as

27 $$\begin{aligned} S^{z}(\mathbf{q})=\sum _{\mathbf{k},\sigma }\sigma \Big ( a^{\dag \sigma }_{\mathbf{k}+\mathbf{q}} a^{\sigma }_\mathbf{k}+b^{\dag \sigma }_{\mathbf{k}+\mathbf{q}} b^{\sigma }_\mathbf{k}\Big ) \end{aligned}$$

Also $\chi _{zz}$ is introduced as longitudinal spin susceptibility and its relation can be expressed in terms of correlation function between *z* component of spin operators as

28 $$\begin{aligned} \chi _{zz}(\mathbf{q},\omega )= & {} i \int _{-\infty }^{+\infty }dt e^{i\omega t} \langle [S^{z}(\mathbf{q},t),S^{z}(-\mathbf{q},0)]\rangle \nonumber \\= & {} \lim _{i\Omega _{n} \longrightarrow \omega +i0^{+}}\int _{0}^{1/(k_{B}T)}d\tau e^{i\Omega _{n}\tau }\langle {\mathcal {T}}S^{z}(\mathbf{q},\tau )S^{z}(-\mathbf{q},0)\rangle \nonumber \\= & {} \chi _{zz}(\mathbf{q},i\Omega _{n}\longrightarrow \omega +i0^{+}). \end{aligned}$$

After some algebraic calculations similar to transverse spin susceptibility case, we arrive te final results for Matsubara representation of longitudinal dynamical spin susceptibility as

29 $$\begin{aligned} \chi _{zz}(\mathbf{q},i\Omega _{n})= & {} - \frac{1}{N}\sum _{\mathbf{k}}\sum _{\alpha ,\beta ,\sigma }\int ^{+\infty }_{-\infty } \int ^{+\infty }_{-\infty }\frac{d\epsilon d\epsilon ^{\prime }}{\pi ^{2}} {\mathrm{ImG}} ^{\sigma }_{\beta \alpha }(\mathbf{k},\epsilon +i0^{+}){\mathrm{ImG}} ^{\sigma }_{\alpha \beta }(\mathbf{k}+\mathbf{q},\epsilon ^{\prime }+i0^{+})\nonumber \\&\times \frac{n_{F}(\epsilon )-n_{F}(\epsilon ^{\prime })}{i\Omega _{n}+\epsilon -\epsilon ^{\prime }}, 
\end{aligned}$$

The dynamical spin structure factor for both longitudinal and transverse spin directions are obtained based on retarded presentation of susceptibilities as

30 $$\begin{aligned} \chi _{zz}(\mathbf{q},\omega )=\chi _{zz}(q,i\Omega _{n}\longrightarrow \omega +i0^+)\;\;,\;\; \chi _{+-}(\mathbf{q},\omega )=\chi _{+-}(q,i\Omega _{n}\longrightarrow \omega +i0^+). \end{aligned}$$

so that $\chi _{zz}$ and $\chi _{+-}$ are retarded dynamical spin structure factors for longitudinal and transverse components of spins, respectively. The imaginary part of retarded dynamical spin structure factor, i.e. ${\mathrm{Im}}\upchi _{+-}(\mathbf{q},\omega ),{\mathrm{Im}}\upchi _{zz}(\mathbf{q},\omega )$, is proportional to the contribution of localized spins in the neutron differential cross-section. For each $\mathbf{q}$ the dynamical structure factor has peaks at certain energies which represent collective excitations spectrum of the system. The imaginary parts of both retarded transverse and longitudinal spin structure factors are given by

31 $$\begin{aligned} {\mathrm{Im}}\upchi _{+-}(\mathbf{q},\omega )= & {} \frac{1}{N}\sum _{\mathbf{k}}\sum _{\alpha ,\beta }\int ^{+\infty }_{-\infty }\frac{d\epsilon }{\pi } {\mathrm{ImG}} ^{\uparrow }_{\beta \alpha }(\mathbf{k},\epsilon +i0^{+}){\mathrm{ImG}} ^{\downarrow }_{\alpha \beta }(\mathbf{k}+\mathbf{q},\epsilon +\omega +i0^{+})\nonumber \\&\times \Big (n_{F}(\epsilon )-n_{F}(\epsilon +\omega )\Big ) ,\nonumber \\ {\mathrm{Im}}\upchi _{zz}(\mathbf{q},\omega )= & {} \frac{1}{N}\sum _{\mathbf{k}}\sum _{\alpha ,\beta ,\sigma }\int ^{+\infty }_{-\infty }\frac{d\epsilon }{\pi } {\mathrm{ImG}} ^{\sigma }_{\beta \alpha }(\mathbf{k},\epsilon +i0^{+}){\mathrm{ImG}} ^{\sigma }_{\alpha \beta }(\mathbf{k}+\mathbf{q},\epsilon +\omega +i0^{+})\nonumber \\&\times \Big (n_{F}(\epsilon )-n_{F}(\epsilon +\omega )\Big ). \end{aligned}$$

The frequencies of collective magnetic excitation modes are determined via finding the position of peaks in imaginary part of of imaginary part of dynamical spin susceptibilities.

Static transverse spin structure factor ($s_{+-}(\mathbf{q})$) which is a measure of magnetic long range ordering for spin components along the plane, i.e. transverse direction, can be related to imaginary part of retarded dynamical spin susceptibility using following relation

32 $$\begin{aligned} s_{+-}(\mathbf{q})= \left\langle S^{+}(\mathbf{q})S^{-}(-\mathbf{q}) \right\rangle= & {} k_{B}T\sum _{n}\frac{1}{2\pi } \int _{-\infty }^{\infty }d\omega \frac{-2{\mathrm{Im}}\upchi _{+-}(\mathbf{q},i\omega _{n}\longrightarrow \omega +i0^+)}{i\omega _{n}-\omega }\nonumber \\= & {} \int _{-\infty }^{+\infty }d\omega \frac{n_{B}(\omega )}{\pi }{\mathrm{Im}}\upchi _{+-} (\mathbf{q},i\omega _{n}\longrightarrow \omega +i0^+). \end{aligned}$$

Moreover can find the static longitudinal spin structure factor, i.e. $s_{+-}(\mathbf{q})$, by using ${\mathrm{Im}}\upchi _{zz}(\mathbf{q},\omega )$ as

33 $$\begin{aligned} s_{zz}(\mathbf{q})= & {} \left\langle S^{z}(\mathbf{q})S^{z}(-\mathbf{q}) \right\rangle = k_{B}T\sum _{n}\frac{1}{2\pi } \int _{-\infty }^{\infty }d\omega \frac{-2{\mathrm{Im}}\upchi _{zz}(\mathbf{q},i\omega _{n}\longrightarrow \omega +i0^+)}{i\omega _{n}-\omega }\nonumber \\= & {} \int _{-\infty }^{+\infty }d\omega \frac{n_{B}(\omega )}{\pi }{\mathrm{Im}}\upchi _{zz} (\mathbf{q},i\omega _{n}\longrightarrow \omega +i0^+). \end{aligned}$$

In the next section, the numerical results of dynamical spin structure and static spin structures of Germanene layer have been presented for various magnetic field and spin-orbit coupling strength.

## Numerical results and discussions

We turn to a presentation of our main numerical results of imaginary part of dynamical structure factors of Germanene layer at finite temperature in the presence of magnetic field and spin-orbit coupling. Also the temperature dependence of static structure factors has been addressed in this section. Using the electronic band structure, the matrix elements of Fourier transformations of spin dependent Green’s function are calculated according to Eq. (19). The imaginary part of both transverse and longitudinal dynamical spin susceptibilities is made by substituting the Green’s function matrix elements into Eq. (31). In the following, the frequency behavior of imaginary part of dynamical spin susceptibilities is studied at fixed wave number $\mathbf{q}=(0,\frac{4\pi }{3})$ in the Brillouin zone where the length of unit cell vector of honeycomb lattice is taken to be unit. Furthermore the static structure factors have been obtained by using Eqs. (32, 33).

The frequency behaviors of both the transverse and longitudinal dynamical spin susceptibilities have been addressed in this present study. Also the spin structure factors behaviors have been investigated for Germanene structure.

The optimized atomic structure of the Germanene with primitive unit cell vector length $a=1$ is shown in Fig. 1. The primitive unit cell include two Ge atoms.

**Figure 3:**
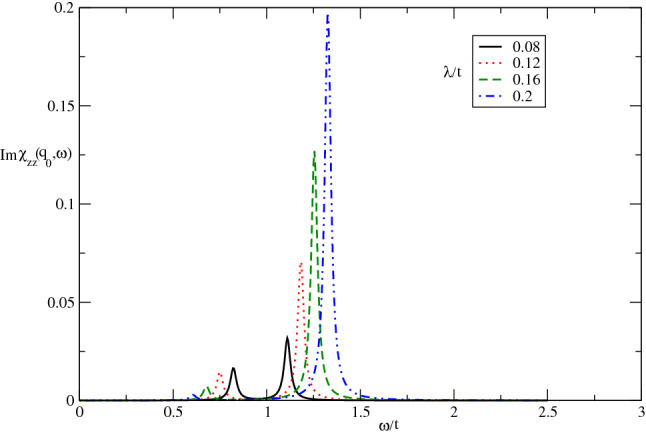
The imaginary part of dynamical longitudinal spin susceptibility ${\mathrm{Im}}\upchi _{zz}(\mathbf{q}_{0},\omega )$ of undoped Germanene layer versus normalized frequency $\omega /t$ for different values of normalized spin-orbit coupling $\lambda /t$ at fixed temperature $k_{B}T/t=0.05$. The magnetic field is considered to be zero.

In Fig. 3, we depict the frequency dependence of imaginary part of longitudinal dynamical spin susceptibility, $Im\chi _{zz}(\mathbf{q}_{0},\omega )$, of undoped Germanene layer for different values of spin-orbit coupling, namely $\lambda /t=0.08,0.12,0.16,0.2$, in the absence of magnetic field by setting $k_{B}T/t=0.05$. In fact the effects of spin-orbit coupling strength on frequency dependence of $Im\chi _{zz}(\mathbf{q},\omega )$ have been studied in this figure. As shown in Fig. 3, the frequency positions of sharp peaks in $Im\chi _{zz}(\mathbf{q}_{0},\omega )$, that imply spin excitation mode for longitudinal components of spins, moves to higher frequencies with increase of spin-orbit coupling. This fact can be understood from this point that the increase of spin-orbit coupling leads to enhance band gap in density of states and consequently the excitation mode appears in higher frequency. Note that this figure shows the inelastic cross section neutron particles from itinerant electrons of the system due to longitudinal component along *z* direction of magnetic moment of electrons and neutron beam. Another novel feature in Fig. 3 is the increase of intensity of sharp peak with $\lambda$. For frequencies above normalized value 1.5, there is no collective magnetic excitation mode for longitudinal components of electron spins as shown in Fig. 3.

**Figure 4:**
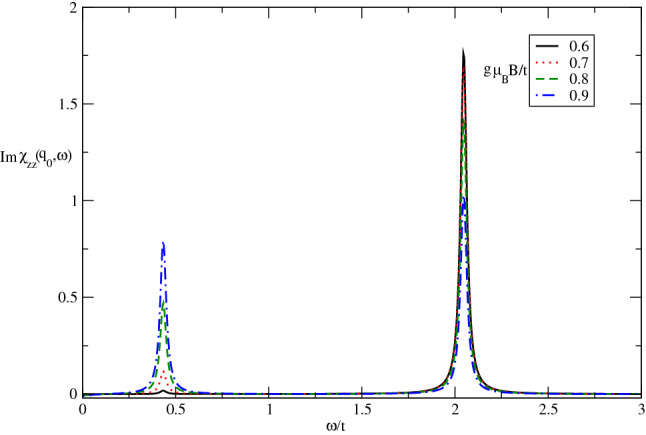
The imaginary part of dynamical longitudinal spin susceptibility ${\mathrm{Im}}\upchi _{zz}(\mathbf{q}_{0},\omega )$ of undoped Germanene layer versus normalized frequency $\omega /t$ for different values of normalized magnetic field $g\mu _{B}B/t$ at fixed spin-orbit coupling $\lambda /t=0.6$. The normalized temperature is considered to be $k_{B}T/t=0.05$.

The frequency dependence of imaginary part of longitudinal dynamical spin structure factor of undoped Germanene layer in the presence of spin polarization for different magnetic field values $g\mu _{B}B/t$ has been shown in Fig. 4 at fixed $\lambda /t=0.6$ by setting $k_{B}T/t=0.05$. A novel feature has been pronounced in this figure. It is clearly observed that all curves for different magnetic field indicates two clear magnetic excitation collective modes at frequencies $\omega /t\approx 0.45$ and $\omega /t\approx 2.1$. The frequency positions of magnetic excitation mode is independent of magnetic field. The intensity of sharp peaks in $\omega /t\approx 2.1$ in imaginary part of longitudinal susceptibility, i.e. $Im\chi _{zz}(\mathbf{q}_{0},\omega )$, decreases with magnetic field. However the height of sharp peaks in frequency position $\omega /t\approx 0.45$ enhances with increase of spin-orbit coupling as shown in Fig. 4.

**Figure 5:**
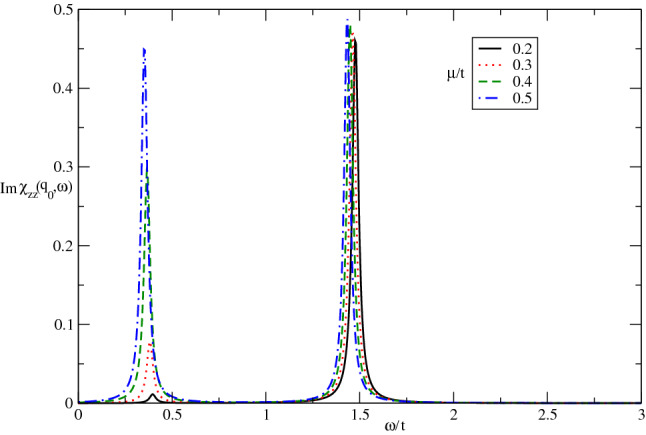
The imaginary part of dynamical longitudinal spin susceptibility ${\mathrm{Im}}\upchi _{zz}(\mathbf{q}_{0},\omega )$ of doped Germanene layer versus normalized frequency $\omega /t$ for different values of normalized chemical potential $\mu /t$ at fixed spin-orbit coupling $\lambda /t=0.3$. The magnetic field is assumed to be zero. The normalized temperature is considered to be $k_{B}T/t=0.05$.

In Fig. 5, the imaginary part of longitudinal dynamical spin structure factor of Germanene layer has been plotted for different values of chemical potential, namely $\mu /t=0.2,0.3,0.4,0.5$, at fixed spin-orbit coupling $\lambda /t=0.3$ by setting $k_{B}T/t=0.05$ in the absence of magnetic field. This figure implies the frequency position and intensity of collective excitation mode in $\omega /t\approx 1.5$ has no dependence on chemical potential. Although the intensity of sharp peak in $Im\chi _{zz}(\mathbf{q}_{0},\omega )$ at frequency position $\omega /t\approx 0.4$ increases with chemical potential. There is no considerable change for frequency position in $Im\chi _{zz}(\mathbf{q}_{0},\omega )$ at $\omega /t\approx 0.4$ with chemical potential according to Fig. 5. Also the intensity of low energy magnetic excitation mode reduces with decreasing chemical potential value $\mu$.

**Figure 6:**
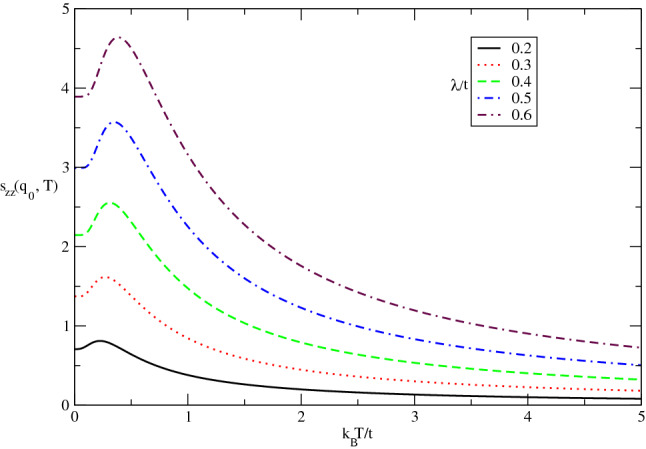
The longitudinal static spin structure factor $s_{zz}(\mathbf{q}_{0},T)$ of undoped Germanene layer versus normalized temperature $k_{B}T/t$ for different values of spin-orbit coupling strength $\lambda /t$ in the absence of magnetic field.

The behavior of longitudinal static spin structure factor $s_{zz}(\mathbf{q}_{0})$ of undoped Germanene layer in terms of normalized temperature $k_{B}T/t$ for different values of $\lambda /t$ has been presented in Fig. 6. The applied magnetic field is assumed to be zero. This function is a measure for the tendency to magnetic long range ordering for the longitudinal components of spins in the itinerant electrons. This figure implies static spin structure for spin components perpendicular to the Germanene plane includes a peak for each value of $\lambda /t$. The temperature position of peak in longitudinal spin static structure factor is the same for all spin-orbit coupling strengths. Although the height of peak increases with $\lambda /t$ which justifies the long range ordering for *z* components of spins improves with spin-orbit coupling. Another novel feature is the non zero value for static structure factor $s_{zz}(\mathbf{q}_{0})$ at zero temperature. However the increase of temperature up to peak position leads to raise magnetic long range ordering. Upon more increasing temperature above normalized value 0.25, the thermal fluctuations causes to reduce $s_{zz}(\mathbf{q}_{0})$ so that magnetic long range ordering of the electrons decays as shown in Fig. 6.

**Figure 7:**
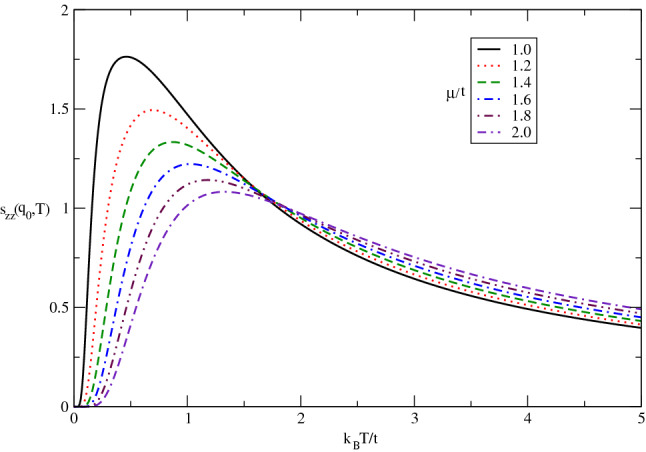
The longitudinal static spin structure factor $s_{zz}(\mathbf{q}_{0},T)$of undoped Germanene layer versus normalized temperature $k_{B}T/t$ for different values of chemical potential $\mu /t$ in the absence of magnetic field. Spin-orbit coupling strength has been fixed at $\lambda /t=0.4$.

The temperature dependence of static longitudinal spin structure factor of doped Germanene layer for various chemical potential has been studied in Fig. 7 for $g\mu _{B}B/t=0.0$ by setting $\lambda /t=0.4$. In contrast to the undoped case in Fig. 6, it is clearly observed the longitudinal spin structure factor for each value finite chemical potential gets zero value at zero temperature according to Fig. 7. In fact the quantum fluctuations at zero temperature for doped case leads to destroy any magnetic long range ordering. Moreover the peak in temperature dependence of $s_{zz}(\mathbf{q}_{0})$ tends to higher temperature upon increasing electron doping. Also the height of peak ,as a measure of magnetic long range ordering for longitudinal components of spins, reduces with chemical potential according to Fig. 7. Upon increasing temperature above normalized value 1.5, $s_{zz}(\mathbf{q}_{0})$ increases with $\mu /t$ and consequently the increase of electron doping improves the long range ordering in temperature region above normalized value 1.5.

**Figure 8:**
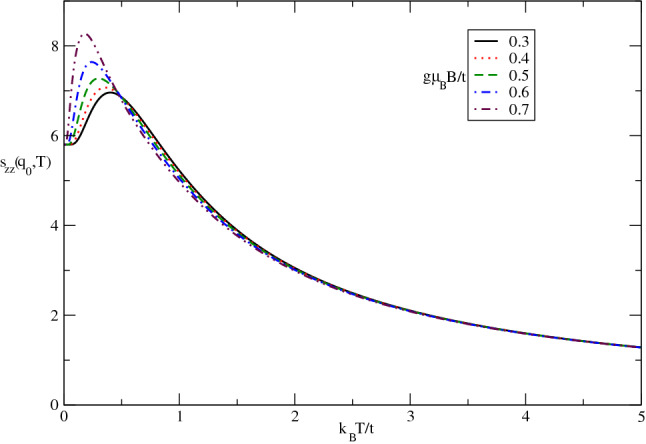
The longitudinal static spin structure factor $s_{zz}(\mathbf{q}_{0},T)$ of undoped Germanene layer versus normalized temperature $k_{B}T/t$ for different values of magnetic field $g\mu _{B}B/t$ at fixed spin-orbit coupling strength $\lambda /t=0.4$.

The effect of longitudinal magnetic field on the behavior of $s_{zz}(\mathbf{q}_{0})$ in terms of normalized temperature $k_{B}T/t$ in undoped case for different values of magnetic field, namely $g\mu _{B}B/t=0.3,0.4,0.5,0.6,0.7$ has been plotted in Fig. 8. The static structure is considerably affected by magnetic field at low temperatures below normalized amount 0.5 where the quantum effects are more remarkable. In addition, at fixed values of temperatures above normalized value 0.5, $s_{zz}(\mathbf{q}_{0})$ is independent of magnetic field and all curves fall on each other on the whole range of temperature in this temperature region. Also temperature position of peak in longitudinal static spin structure factor moves to lower temperature with increasing magnetic field according to Fig. 8. Moreover the height of peak enhances with magnetic field. It can be understood from the fact that applying magnetic field along *z* direction perpendicular to the plane causes long range ordering of *z* components of spins of electrons which increases the longitudinal static structure factor at low temperatures below 0.5.

**Figure 9:**
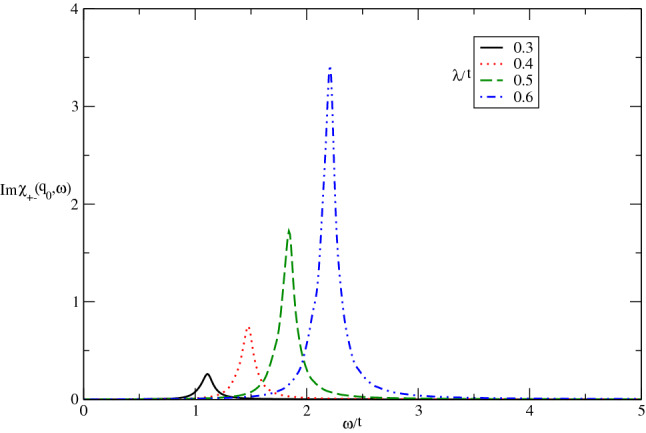
The imaginary part of dynamical transverse spin susceptibility ${\mathrm{Im}}\upchi _{+-}(\mathbf{q}_{0},\omega )$ of undoped Germanene layer versus normalized frequency $\omega /t$ for different values of normalized spin-orbit coupling $\lambda /t$ at fixed temperature $k_{B}T/t=0.05$. The magnetic field is considered to be zero.

We have also studied the dynamical and static transverse spin structure factors of Germanene layer in the presence of magnetic field and spin-orbit coupling. Our main results for imaginary part of dynamical transverse spin susceptibility of undoped Germanene layer for different spin-orbit coupling strengths at fixed temperature $k_{B}T/t=0.05$ in the absence of magnetic field are summarized in Fig. 9. The collective magnetic excitation modes for spin components parallel to the plane tends to higher values with $\lambda /t$ according to Fig. 9. This feature arises from the increase of band gap with spin-orbit coupling so that collective mode appears at higher frequencies as shown in Fig. 9. Also the height of peak in ${\mathrm{Im}}\upchi _{+-}(\mathbf{q}_{0},\omega )$, which is proportional to intensity of scattered neutron beam from itinerant electrons of Germanene structure, reduces with decreasing spin-orbit coupling strength.

**Figure 10:**
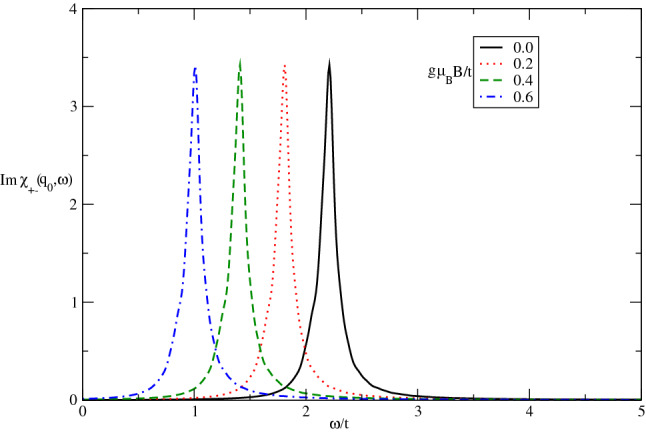
The imaginary part of dynamical transverse spin susceptibility ${\mathrm{Im}}\upchi _{+-}(\mathbf{q}_{0},\omega )$ of undoped Germanene layer versus normalized frequency $\omega /t$ for different values of normalized magnetic field $g\mu _{B}B/t$ at fixed spin-orbit coupling $\lambda /t=0.6$. The normalized temperature is considered to be $k_{B}T/t=0.05$.

In Fig. 10 we plot the numerical results of ${\mathrm{Im}}\upchi _{+-}(\mathbf{q},\omega )$ of undoped Germanene layer as a function of normalized frequency $\omega /t$ for various magnetic field, namely $g\mu _{B}B/t=0.0,0.2,0.4,0.6$ by setting $k_{B}T/t=0.05$. It is clearly observed that the frequency position of collective magnetic excitation mode moves to lower values with magnetic field. It can be understood from the fact that the applying magnetic field gives rise to reduce the band gap and consequently the excitation mode takes place at lower frequencies. Moreover the intensity of collective excitation mode for transverse components of spins is clearly independent of magnetic field strength according to Fig. 10.

**Figure 11:**
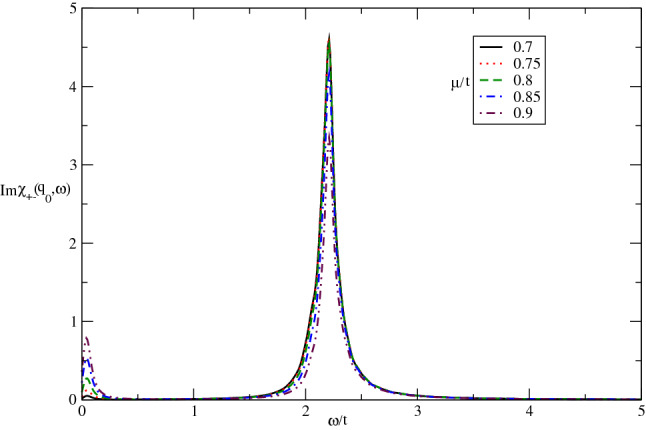
The imaginary part of dynamical transverse spin susceptibility ${\mathrm{Im}}\upchi _{+-}(\mathbf{q}_{0},\omega )$ of doped Germanene layer versus normalized frequency $\omega /t$ for different values of normalized chemical potential $\mu /t$ at fixed spin-orbit coupling $\lambda /t=0.3$. The magnetic field is assumed to be zero. The normalized temperature is considered to be $k_{B}T/t=0.05$.

Figure 11 presents the effect of electron doping on the frequency dependence of ${\mathrm{Im}}\upchi _{+-}(\mathbf{q}_{0},\omega )$ of Germanene layer by setting $\lambda /t=0.3$ at fixed value of temperature in the absence of magnetic field. There are two collective magnetic excitation mode for each value chemical potential. The frequency positions of excitation mode are the same for all values of chemical potential. The intensity of low frequency peak increases with chemical potential however the intensity of high frequency peak reduces with electron doping as shown in Fig. 11. The increase of electron doping leads to decrease transition rate of electron from valence band to conduction one and consequently the intensity of excitation mode decreases.

**Figure 12:**
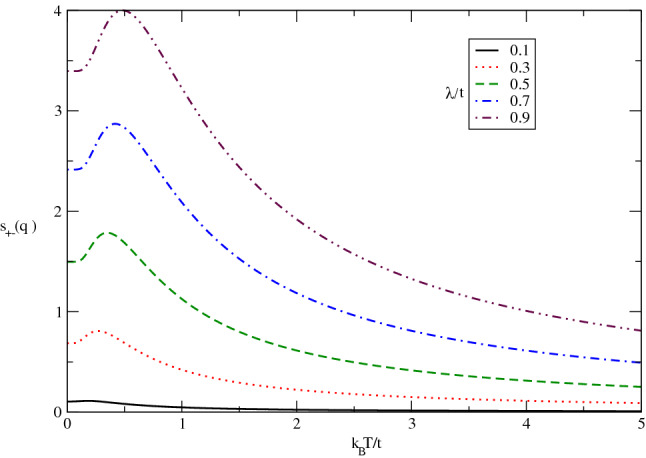
The transverse static spin structure factor $s_{+-}(\mathbf{q}_{0},T)$ of undoped Germanene layer versus normalized temperature $k_{B}T/t$ for different values of spin-orbit coupling strength $\lambda /t$ in the absence of magnetic field.

The behavior of transverse static spin structure factor $s_{+-}(\mathbf{q}_{0})$ of undoped Germanene layer as a function of normalized temperature $k_{B}T/t$ for different values of $\lambda /t$ in the absence of magnetic field has been presented in Fig. 12. This function is a measure for the tendency to magnetic long range ordering for the transverse components of spins in the itinerant electrons. A peak in $s_{+-}(\mathbf{q}_{0})$ is clearly observed for each value of $\lambda /t$. The peak is located at 0.25 for all values of spin-orbit coupling strengths. Although the height of peak increases with $\lambda /t$ which justifies the long range ordering for transverse components of spins improves with spin-orbit coupling. Another novel feature is the non zero value for static structure factor $s_{+-}(\mathbf{q}_{0})$ at zero temperature. In temperature region below peak position, the increase of temperature leads to raise magnetic long range ordering. Upon more increasing temperature above normalized value 0.25, the thermal fluctuations causes to reduce $s_{zz}(\mathbf{q}_{0})$ and magnetic long range ordering of the electrons as shown in fig.(12).

**Figure 13:**
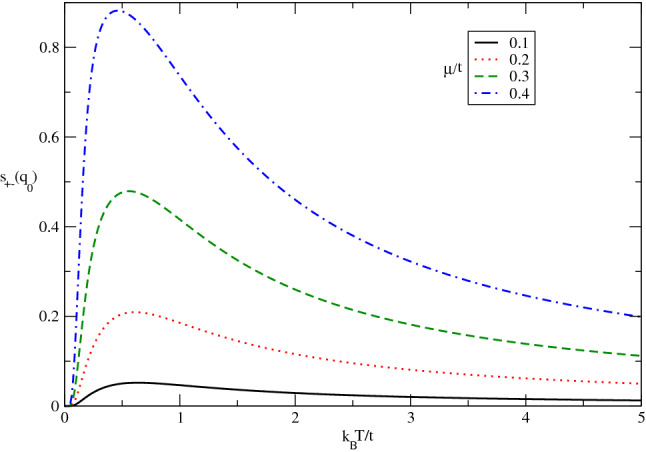
The transverse static spin structure factor $s_{+-}(\mathbf{q}_{0},T)$ of doped Germanene layer versus normalized temperature $k_{B}T/t$ for different values of chemical potential$\mu /t$ in the absence of magnetic field with spin-orbit coupling $\lambda /t=0.3$.

The temperature dependence of static transverse spin structure factor of doped Germanene layer for various chemical potential in the absence of magnetic field has been studied in Fig. 13 by setting $\lambda /t=0.3$. In contrast to the undoped case in Fig. 12, it is clearly observed the transverse spin structure factor for each value finite chemical potential gets zero value at zero temperature according to Fig. 13. In fact the quantum fluctuations at zero temperature for doped case leads to destroy any magnetic long range ordering. Moreover the temperature position of peak in $s_{+-}(\mathbf{q}_{0})$ appears in $k_{B}T/t=0.5$ for all amounts of chemical potential. Also the height of peak ,as a measure of magnetic long range ordering for transverse components of spins, reduces with chemical potential according to Fig. 13. In other words the increase of electron doping leads to decrease magnetic long range ordering for transverse components of spins.

**Figure 14:**
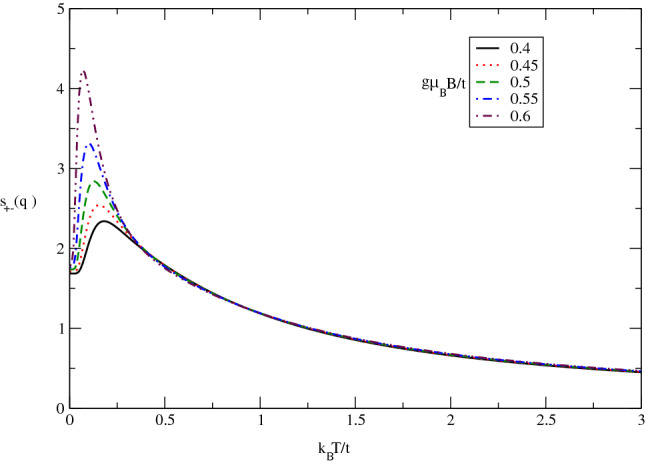
The transverse static spin structure factor $s_{+-}(\mathbf{q}_{0},T)$of undoped Germanene layer versus normalized temperature $k_{B}T/t$ for different values of magnetic field $g\mu _{B}B/t$. Spin-orbit coupling strength has been fixed at $\lambda /t=0.4$.

The effect of longitudinal magnetic field on the behavior of $s_{+-}(\mathbf{q}_{0})$ in terms of normalized temperature $k_{B}T/t$ in undoped case for different values of magnetic field, namely $g\mu _{B}B/t=0.4,0.45,0.5,0.55,0.6$ has been plotted in Fig. 14. The static transverse spin structure factor is considerably affected by magnetic field at low temperatures below normalized amount 0.25 where the quantum effects are more remarkable. In addition, at fixed values of temperatures above normalized value 0.25, $s_{+-}(\mathbf{q}_{0})$ is independent of magnetic field and all curves fall on each other on the whole range of temperature in this temperature region. Also temperature position of peak in longitudinal static spin structure factor moves to lower temperature with increasing magnetic field. Moreover the height of peak enhances with magnetic field. It can be understood from the fact that applying magnetic field along *z* direction perpendicular to the plane causes long range ordering of components of spins parallel to the plane of electrons which increases the longitudinal static structure factor at low temperatures below 0.25.
